# Cardiovascular calcification in hemodialysis patients: A Qatar-based prevalence and risk factors study

**DOI:** 10.5339/qmj.2024.18

**Published:** 2024-03-14

**Authors:** Tarek Ghonimi, Abdullah Hamad, Tarek Fouda, Fadwa AlAli, Mohamad Ezzat, Mohamed Awad, Rania Ibrahim, Mohamed Amin, Mohamad Alkadi, Hassan Ali Al-Malki

**Affiliations:** 1 Division of Nephrology, Department of Medicine, Hamad Medical Corporation, Doha, Qatar Email: abdhamad@hotmail.com

**Keywords:** Bone mineral disease, calcifications, ESRD, dialysis

## Abstract

Background: Patients with end-stage kidney disease on hemodialysis (HD) have an increased risk of death due to the high prevalence of cardiovascular disease. Vascular calcification (VC) is predictive of cardiovascular disease and mortality. We conducted a study to evaluate the prevalence and risk factors for VC in dialysis patients in Qatar.

Methods: This is a retrospective nationwide study including all chronic ambulatory dialysis patients in Qatar from 2020 to 2022. We used our national electronic medical record to track demographics, clinical characteristics, comorbidities, laboratory values, and diagnostic data for each patient. Calcifications were assessed by echocardiography (routinely done for all our dialysis population per national protocol), computed tomography, X-ray, and ultrasound. The study protocol was approved by the local medical research ethics committee (MRC-01-20-377).

Results: 842 HD patients were included in this study. Vascular calcifications (VC) were prevalent in 52.6% of patients. The main site of VC was Mitral valve calcifications in 55.5% of patients. Patients with VC were significantly older and had more prevalence of diabetes mellitus (*p* = 0.001 and *p* = 0.006, respectively). There was no statistically significant difference between patients with calcifications and patients without calcifications regarding serum calcium, phosphorus, and PTH level. In multivariate analysis, age and diabetes significantly increased the risk factor for calcification (95% CI 1.033–1.065, *p* < 0.0001, and 95% CI 1.128–2.272, *p* < 0001, respectively). Moreover, higher vitamin D levels and higher doses of IV Alfacalcidol were significant risk factors for calcifications (95% CI 1.005–1.030, *p* < 0.007, and 95% CI 1.092–1.270, *p* < 0.0001, respectively).

Conclusion: Our study found that vascular calcification was widespread among our dialysis population in Qatar. Implementing the practice of echocardiography in dialysis patients was extremely helpful and the most productive in detecting vascular calcification. Diabetes mellitus almost doubles the risk for vascular calcifications in dialysis patients. These results are beneficial in identifying risk factors for vascular calcification, which can help stratify dialysis patients’ risk of cardiovascular disease and optimize prevention efforts.

## Background

End-stage kidney disease (ESRD) is a prevalent and debilitating condition affecting millions of individuals worldwide, characterized by a near-total loss of renal function. Among the numerous complications associated with ESRD, vascular calcification has emerged as a significant concern due to its association with cardiovascular disease (CVD) morbidity and mortality.^1^ Vascular calcification involves the pathological deposition of calcium phosphate crystals within the walls of arteries and veins, leading to vessel stiffness, decreased compliance, and an increased risk of adverse cardiovascular events such as myocardial infarction and stroke.^2^ Vascular calcification is highly prevalent in ESRD patients and occurs decades earlier than in the general population,^3^ and its progression accelerates dramatically once a patient initiates chronic dialysis.^4^ This is of great clinical significance, as the presence and degree of calcification independently predict future cardiovascular events, as well as mortality.^5,6^

Calcification can occur in both the intimal and medial layers of the vasculature, but medial calcification is considered the more common and significant form of calcification in ESRD.^7^

Aortic arterial calcification (AAC) increases with age^8^ and has been associated with an increased risk of cardiovascular incidents.^9,10^ The presence of arterial calcification is predictive of CVD and mortality.^10^ AAC is an independent predictor of obstructive coronary disease and correlates with coronary artery calcification (CAC).^11^ Up to 80–90% prevalence of vascular calcification in ESRD patients has been reported.^12^ Coronary calcification is associated with CVD, myocardial infarction, and all-cause mortality in the CKD population, including patients on dialysis.^13^ Bone mineral metabolism is tightly regulated by a delicate balance between parathyroid hormone (PTH), fibroblast growth factor 23 (FGF23), vitamin D, and other factors. In ESRD patients, disturbances in this equilibrium occur due to impaired renal excretion of phosphate and reduced vitamin D activation, leading to secondary hyperparathyroidism, elevated serum phosphate levels, and abnormal FGF23 concentrations.^14^ These derangements in mineral metabolism have been linked to the development and progression of vascular calcification in ESRD patients.^15^ Hemodialysis (HD), a cornerstone therapy for ESRD, often further exacerbates the risk of vascular calcification through mechanisms that involve alterations in bone mineral metabolism.^16^ This intricate interplay between bone and vasculature has gained increasing recognition in recent years, with research focusing on elucidating the underlying pathophysiological mechanisms, assessing its consequences on patients’ health, and exploring the best modalities in monitoring and treatment.^17^

Hamad Medical corporation (HMC), a network of government hospitals, is the sole provider of Hemodialysis (HD). Our healthcare system consists of inpatient and ambulatory dialysis services. All ambulatory dialysis units under Hamad General Hospital were included in this study (4 dialysis facilities: Fahad Bin Jassim Kidney Center, Al Shamal, Al Shahaniya, and Al Wakra units). Details of our service have been explained in multiple publications.^18,19^ Dialysis populations are rising in the State of Qatar and are predicted to rise 5% annually till 2030. Projections indicate that the number of dialysis patients being cared for at any given time will surpass 1600 by that year.^20^

The objective of this study is to evaluate the possible relationship between vascular calcification and bone mineral metabolism in patients on hemodialysis.

## Methods

This is a retrospective nationwide study conducted in the ambulatory dialysis units in Qatar from 2020 to 2022.

### Study Subjects

#### Inclusion Criteria

We included patients with end-stage kidney disease receiving chronic hemodialysis treatment in the period between January 2020 and December 2022. The patient should be on continuous hemodialysis for more than 3 months. The patient should have regular biochemical laboratory tests for mineral and bone disorder (MBD) done per unit protocol.

#### Exclusion Criteria

We excluded pregnant female patients, patients lacked blood tests related to MBD, and children (under the age of 14 years).

### Study Procedure and Data Collection

We utilized our electronic medical record system to monitor and record various patient-related information, such as demographics, clinical characteristics, comorbidities, laboratory values, and diagnostic data. The foundational demographic data encompassed age and gender, while comorbidities were assessed in terms of the presence of conditions such as diabetes mellitus, hypertension, cardiovascular disease, cerebrovascular disease, obstructive lung disease, chronic liver disease, and malignancy. Laboratory investigations included parathyroid hormones (PTH), serum-corrected calcium, and phosphorus, and vitamin D levels were recorded for all patients in the study.

Moreover, all data for medications used for bone and mineral disorder were also collected (calcium carbonate, Sevelamer carbonate, Intravenous Alfacalcidol, Cinacalcet and Intravenous Etelcalcetide) and the average medication doses per week were calculated.

Vascular calcifications were assessed by echocardiography (which is routinely done for all our dialysis population per local protocol). Other radiology studies (that were done for other indications during the study period) were used to detect vascular calcification too. Investigations used included abdominal X-ray, chest X-ray, abdominal and pelvis computed tomography (CT), abdominal and extremities ultrasound, neck ultrasound (US) Doppler, and others. The severity of vascular calcification was reported if it was included in the radiology report.

### Statistical Analysis

Statistical analysis was performed using Statistical Package for Social Sciences version 17.0 for Windows (SPSS Inc., Chicago, IL, USA). Normally distributed data were expressed as mean ± standard deviation or median and range, while categorical variables were expressed as absolute and relevant frequencies. For comparison between groups, a paired t-test was used for normally distributed variables, while a chi-square test was used for ordinal distributed variables. *P* < 0.05 (two-tailed) was considered statistically significant. Multivariate regression analysis was done to assess the risk factors for vascular calcifications.

### Ethical Approval

This study was approved by the Institutional Review Board (IRB), Medical Research Center of Hamad Medical Corporation (MRC-01-20-377).

## Results

### 1- Study Population and Demographics

842 HD patients fulfilled the inclusion criteria and were included in the study. The mean age was 59.8 ± 15.3 years, and 61.8% were male, while 38.2% were female. 56.6% of the patients were native population while 43.4% were expatriates. The most common comorbidities were hypertension in 96.3% of patients, followed by diabetes in 69.2% of patients (Table 1).

Calcifications were prevalent in 52.6% of the patients. Most calcifications (63.6%) were detected by cardiac echocardiogram as it is done routinely for all patients in our unit, CT scan of different reasons/areas (24.3%), and X-ray of different areas and reasons (11.5%). The main sites of calcifications were Mitral valve calcifications (55.5% of patients), vascular calcification in other arterial vessels (19.9%), and aortic calcification (14.9). For the aortic calcifications, the main site was the abdominal aorta (57.6%) followed by the aortic valve (24.2%). Furthermore, 51.9% of calcification was mild, while 16.4% was moderate and 4.9% was severe. The severity of vascular calcification was reported on most radiology imaging reports (about 80%). In patients with VC, the majority of the patients had mild calcifications (50%), but 35% of the patients had severe calcifications.

The mean PTH level was 408.3 ± 292.3 pg/mL, and the median was 355.4 pg/mL with interquartile values (25%, 227.4 pg/mL, 75%, 457.6 pg/mL). Mean values for calcium, phosphorus, and vitamin D were within the target range for dialysis patients. Most of the patients (85.1%) were using phosphate binders. Sevelamer was the most prevalent (395 patients 55%) with an average weekly dose of 25,631 mg (average daily number of tablets of 4.6), followed by Calcium Carbonate (322 patients, 45%), with a mean weekly dose of 8358 mg (daily dose of 2.2 tablets). Table 1 summarizes the demographic and general characteristics of the study population.

### 2- Comparison Between Patients with Vascular Calcification and Without Vascular Calcifications

Patients with vascular calcifications were significantly older (61.6y/o vs. 58 y/o *p* = 0.001) and were more likely to have diabetes mellitus compared to patients without vascular calcifications (75% vs. 28.6% *p* = 0.006). There was no statistically significant difference between patients with vascular calcifications and patients without it regarding serum calcium and PTH levels (*p* = 0.4 and *p* = 0.75, respectively). However, Patients with calcifications had significantly higher vitamin D levels and lower phosphorus levels than patients without calcifications (*p* = 0.003 and *p* = 0.018, respectively) (Table 2).

The weekly doses of calcium carbonate and Etelcalcetide showed no significant differences between the two groups (*p* = 0.758 and *p* = 0.327, respectively). However, there were significantly higher doses of cinacalcet and IV Alfacalcidol in patients with calcifications than in patients without calcifications (*p* = 0.021 and *p* = 0.003, respectively) (Table 2).

### 3- Regression Analysis: Risk Factors For Vascular Calcifications in ESRD Patients on Hemodialysis

#### A- Univariate Analysis

Parameters with significant differences between patients with VC and no VC were further analyzed. In univariate analysis, we found that all factors significantly different in the previous analysis carried a statistically significant risk for VC (DM, Age, Vitamin D level, Cinacalcet dose, and IV Alfacalcidol dose). The strongest risk was for DM with OR 2.65 (1.958–3.595, *P* = 0.000) (Table 3).

#### B- Multivariate Analysis

Diabetes carried the most significant risk for vascular calcification (odds 1.768, 95 CI% 1.128–2.272, *P* < 0001). Age was also significant but less risky (odds 1.049, 95% CI 1.033 = 1.065, *p* < 0.0001). Moreover, higher vitamin D levels and higher doses of IV Alfacalcidol were statistically significant risk factors for calcifications (odds 1.017. 95% CI 1.005 = 1.030 *p* < 0.007 and odds 1.178 95% CI 1.092 = 1.270, *p* < 0.0001, respectively). Although oral cinacalcet dose was a weak risk factor in the univariate analysis, it became statistically in significant in the univariate analysis with *p* < 0.051 (Table 3).

## Discussion

Vascular calcification poses a significant challenge among patients with end-stage kidney disease (ESRD), as it is intricately linked with unfavorable outcomes.^21^ Despite its global relevance, there is a notable paucity of research specifically addressing this issue in Qatar. Our study, the first of its kind in the region, delves into the prevalence of vascular calcifications in the hemodialysis population and scrutinizes potential risk factors through a comprehensive multivariate analysis.

The Kidney Disease Improving Global Outcomes (KDIGO) CKD-MBD Working Group guidelines for CKD-MBD 2017^22^ propose that lateral abdominal radiograph can be used to detect the presence or absence of vascular calcification and an echocardiogram can be used to detect the presence or absence of valvular calcification as an alternative to computed tomography-based imaging. Patients with CKD and known prevalent vascular or valvular calcification should be treated as the highest category of cardiovascular risk, and this should guide the management of CKD–MBD.

Over the past decade, research publications have consistently reported a prevalence of vascular calcification within the dialysis population ranging from 60% to 90%. The variation in these figures can be attributed to factors such as the geographical region under study and the diagnostic methods employed. For instance, a study by London et al. in France, which included 202 hemodialysis patients with a dialysis regimen comparable to ours, revealed a 68% prevalence of vascular calcifications. The diagnostic approach in this study involved the combined use of X-ray and echocardiogram, aligning with the alternative methods recommended by the Kidney Disease Improving Global Outcomes (KDIGO) guidelines.^23^ Highlighting the global relevance of vascular calcification, a study conducted in Australia involving 137 hemodialysis patients reported a substantial 90% prevalence of abdominal aortic vascular calcification.^24^

Our study included 842 patients on regular hemodialysis. Vascular calcification’s traditional risk factors include advanced age, hypertension, and diabetes. Non-traditional risk factors in CKD include disorders of mineral metabolism, elevated serum PTH levels, excessive intake of calcium supplements, inflammation, malnutrition, and oxidative stress.^25^

In our study, we found that the presence of diabetes mellitus almost doubled the risk of vascular calcifications, Table 2. Vascular calcifications are more prevalent and more advanced in patients with diabetes mellitus compared to non-diabetics. Chronic kidney disease (especially end-stage kidney disease and dialysis) increases that risk significantly.^26,27^ According to Ishimura et al.,^28^ the prevalence of arterial calcification in people with diabetes was 47.1%, compared with 13.6% in non-diabetics.^29,30^ Thet semi-quantitatively examined abdominal aortic calcification (mostly considered to be intimal calcification) of hemodialysis patients using computed tomography and demonstrated that the degree of abdominal aortic calcification was significantly advanced in people with diabetes compared to non-diabetics. Old age was the most significant factor in vascular calcification.^31–34^ Old age was associated with vascular calcification in both our study and its predecessors.^35,36^

In our study, we observed no association between the levels of calcium, phosphorus, and PTH and the occurrence of calcification. However, our findings notably revealed a significant correlation between elevated vitamin D levels and vascular calcification (Tables 2 and 3). Our findings are similar to those of another study by Rosa-Diez et al.,^37^ while London et al. observed that in adult hemodialysis patients, 1,25D and 25D levels were negatively correlated with aortic stiffness but not with vascular calcification.^38^

Our study stands out for being the inaugural investigation into the dialysis population in Qatar to explore vascular calcifications in this high-risk population. Conducted on a nationwide scale, our study utilized data sourced from the national electronic medical record, recognizing that while this method ensures a comprehensive dataset, the possibility of occasional missing data is acknowledged. Our practice mandates having an echocardiogram in all dialysis patients elevating the precision and thoroughness of vascular calcification identifications in this patient population.

### Study Limitations

Our study has some limitations, first, its retrospective nature, although this was mitigated by the comprehensive national Electronic Medical Record (EMR), which ensured the availability and accuracy of all required data. Additionally, the methods for detecting calcifications were not uniformly applied across all patients, except echocardiogram. However, employing varied methods for vascular calcification detection contributed to the overall accuracy of the data. Furthermore, our study is constrained by the absence of dialysis vintage (time spent by patients on dialysis) data within the studied population. This limitation poses challenges in comprehensively assessing the potential risk factors for calcifications, particularly among younger patients. Recognizing the significance of dialysis vintage as a variable, its absence underscores the need for cautious interpretation of our findings. Future studies incorporating this crucial parameter would provide a more nuanced understanding of the relationship between dialysis vintage and the risk of calcifications.

While acknowledging the limitations of our study, we recommend implementing a standardized approach for detecting calcifications in all dialysis patients, ensuring uniformity in diagnostic methods. This could involve incorporating more comprehensive imaging techniques beyond echocardiogram, thereby enhancing the overall accuracy of vascular calcification assessments. Future research may benefit from prospective designs to further investigate and address the nuances associated with calcification detection in this patient population.

## Conclusions

Our national study is the first to report a high prevalence of vascular calcifications in the dialysis population in Qatar. Implementing the mandatory practice of having echocardiography in dialysis patients was extremely helpful and productive in detecting vascular calcification. Diabetes mellitus almost doubles the risk of vascular calcifications in dialysis patients. Old age, higher vitamin D levels, and high doses of IV Alfacalcidol were significant risk factors for calcifications in dialysis patients. These results are beneficial in identifying risk factors for vascular calcification, which can help stratify dialysis patients’ risk of cardiovascular disease and optimize prevention efforts. Considering these findings, we recommend implementing a standardized protocol for vascular calcification screening in dialysis patients, ensuring consistent and thorough assessments. Additionally, healthcare providers should be vigilant in monitoring and managing vascular calcification risk factors, with particular attention to diabetes mellitus, advanced age, and vitamin D levels. Further research is warranted to explore preventive interventions tailored to individuals at higher risk.

## Abbreviations

HD, hemodialysis; VC, vascular calcification; ESRD, end-stage kidney disease; CVD, cardiovascular disease; AAC, aortic arterial calcification; CAC, coronary artery calcification; PTH, parathyroid hormone; FGF23, fibroblast growth factor 23; MBD, mineral and bone disorder; KDIGO, kidney disease improving global outcomes.

## Competing of Statement

We declare that none of the authors have competing financial or non-financial interests.

## Data Availability

All data are fully available without restriction up on request.

## Contribution Statement

We certify that we have participated sufficiently in the intellectual content, conception, and design of this work or the analysis and interpretation of the data (when applicable), as well as the writing of the manuscript, to take public responsibility for it and have agreed to have our name listed as a contributor. We certify that all the data collected during the study are presented in this manuscript, and no data from the study have been or will be published separately. We attest that we will provide the data if requested according to the HMC policy. Financial interests, direct or indirect, that exist or may be perceived to exist for individual contributors in connection with the content of this paper have been disclosed in the cover letter.

## Acknowledgments

We profusely thank S. Aly, A. Jamil, A. Alakhras, A. Shaheen, H. Ateya, and Y. Zitouni for their devotion and dedication in care of patient under Bone and Minerals speciality.

## Figures and Tables

**Table 1.** Patient demographics and laboratory characteristics.

Variable*	**Total Numbers = 842 Mean ± SD, *N* (%)**
Age/year	59.8 ± 15.3
Ethnicity:	
Native/expatriate	476/366 (56.6/43.4)
Gender	
Male/Female	520/322 (61.8/38.2)
Comorbidities:	
Hypertension	815 (96.8)
Diabetes	583 (69.2)
Cardiovascular diseases	459 (54.5)
Malignancy	48 (5.7)
Cerebrovascular disease	70 (8.3)
COPD	60 (7.1)
Chronic liver disease	44 (5.2)
Calcifications:	443 (52.6)
The severity of calcifications:	
Mild	230 (51.9)
Moderate	73 (16.4)
Severe	22 (4.9)
Not applicable	118 (26.6)
Sites of calcifications:	
- Mitral valve	246 (55.5)
- Aorta	66 (14.9)
- Other arterial vascular	88 (19.9)
- Coronary artery	4 (0.9)
- others	39 (8.8)
Site of aortic calcification:	
Aortic valve	16 (24.2)
Aortic arch	12 (18.2)
Abdominal aorta	38 (57.6)
PTH pg/ml	411.6 ± 312.5
corrected calcium mmol/l	2.2 ± 0.17
phosphorus level mmol/l	1.57 ± 0.62
vitamin D nmol/l	37.3 ± 17.6
Calcium carbonate dose mg/week	8358.3 ± 689.3
Sevelamer carbonate dose mg/week	25631.9 ± 14648
Cinacalcet dose mg/week	152.7 ± 138.7
Alfacalcidol dose mcg/week	4.5 ± 2.1
Etelcalcetide dose mcg/week	19.2 ± 6.6

* All values are presented as means + standard deviation (SD). COPD, chronic obstructive pulmonary disease; PTH, parathyroid hormone.

**Table 2.** Comparison between patients with and without calcifications.

Parameters*	**Calcification Number = 433 Mean ± SD, *N* (%)**	**No calcification Number = 409 Mean ± SD, *N* (%)**	**P**
Age/year	61.6 ± 14.2	58.0 ± 16.3	0.001
Gender			
Male/Female	264/177	254/141	0.187
Comorbidities:			
Hypertension	433	397	0.087
DM	325	117	0.006
CVD	248	194	0.393
M align ancy	27	413	0.568
CVA	37	405	0.782
COPD	39	402	0.083
CLD	19	823	0.284
PTH ng/l	408.3 ± 292.3	415.3 ± 335	0.750
corrected calcium mmol/l	2.2 ± 0.17	2.2 ± 0.16	0.400
phosphorus level mmo/l	1.52 ± 0.74	1.63 ± 0.44	0.018
vit D level nmol/l	39.0 ± 18.0	35.4 ± 16.9	0.005
Calcium Carbonate dose mg/week	8,454 ± 6746.1	8,257.1 ± 7065.1	0.758
Sevelamer dose mg/week	25,055.3 ± 13990.0	26,585.2 ± 15477.2	0.384
Oral cinacalcet mg/week	166.0 ± 155.0	133.8 ± 105.5	0.021
IV alfacalcidol mcg/week	4.8 ± 2.3	4.0 ± 1.8	0.003
IV etelcalcetide mcg/week	18.9 ± 6.8	19.8 ± 6.2	0.327

* All values presented as means + standard deviation (SD). DM, Diabetes Mellitus; CVD, Cardiovascular disease; CVA, Cerebrovascular disease; COPD, Chronic obstructive pulmonary disease; CLD, Chronic liver disease.

**Table 3.** Regression analysis: Risk factors for vascular calcifications in ESRD patients on hemodialysis.

	**Univariate Analysis**			**Multivariate Analysis**		
	**odds**	**95% CI**	**p**	**odds**	**95% CI**	**p**
Age	1.054	1.042-1.065	0.000	1.049	1.033-1.065	< 0.0001
Diabetes	2.653	1.958-3.595	0.000	1.768	1.128-2.772	0.013
Vitamin D level	1.013	1.004-1.021	0.002	1.017	1.005-1.030	0.007
Phosphorus	0.634	0.441-0.912	0.014	1.101	0.793-1.528	0.567
Oral Cinacalcet dose	1.002	1.002-1.004	0.000	1.002	1.000-1.003	0.051
IV alfacalcidol dose	1.172	1.096-1.252	0.000	1.178	1.092-1.270	< 0.0001

## References

[1] Ohtake T, Kobayashi S (2017;). Impact of vascular calcification on cardiovascular mortality in hemodialysis patients: clinical significance, mechanisms and possible strategies for treatment. Ren Replace Ther.

[2] Demer LL, Tintut Y (2008;). Vascular calcification: pathobiology of a multifaceted disease. Circulation.

[3] Goodman WG, Goldin J, Kuizon BD, Yoon C, Gales B, Sider D (2000;). Coronary-artery calcification in young adults with endstage renal disease who are undergoing dialysis. N Engl J Med.

[4] Blacher J, Guerin AP, Pannier B, Marchais SJ, London GM (2001;). Arterial calcifications, arterial stiffness, and cardiovascular risk in end-stage renal disease. Hypertension.

[5] London GM, Guerin AP, Marchais SJ (2003;). Arterial media calcification in end-stage renal disease: impact on all-cause and cardiovascular mortality. Nephrol Dial Transplant.

[6] Kauppila LI, Polak JF, Cupples LA, Hannan MT, Kiel DP, Wilson PW (1997;). New indices to classify location, severity and progression of calcifc lesions in the abdominal aorta: a 25-year follow-up study. Atherosclerosis.

[7] Giachelli CM (2009;). The emerging role of phosphate in vascular calcification. Kidney Int..

[8] Wilson PW, Kauppila LI, O’Donnell CJ, Kiel DP, Hannan M, Polak JM (2001;). Abdominal aortic calcifc deposits are an important predictor of vascular morbidity and mortality. Circulation.

[9] van der Meer IM, Bots ML, Hofman A, del Sol AI, van der Kuip DA, Witteman JC (2004;). Predictive value of noninvasive measures of atherosclerosis for incident myocardial infarction: the Rotterdam study. Circulation.

[10] Blacher J, Guerin AP, Pannier B, Marchais SJ, London GM (2001;). Arterial calcifcations, arterial stifness, and cardiovascular risk in end-stage renal disease. Hypertension.

[11] Cho A, Jung HY, Park HC, Oh J, Kim J, Lee YK (2020;). Relationship between abdominal aortic calcifcation on plain radiograph and coronary artery calcifcation detected by computed tomography in hemodialysis patients. Clin Nephrol.

[12] Toussaint ND, Lau KK, Strauss BJ, Polkinghorne KR, Kerr PG (2009;). Determination and validation of aortic calcifcation measurement from lateral bone densitometry in dialysis patients. Clin J Am Soc Nephrol.

[13] Braun J, Oldendorf M, Moshage W, Heidler R, Zeitler E, Luft FC (1996;). Electron beam computed tomography in the evaluation of cardiac calcifcation in chronic dialysis patients. Am J Kidney Dis.

[14] Block GA, Hulbert-Shearon TE, Levin NW, Port FK (1998;). Association of serum phosphorus and calcium x phosphate product with mortality risk in chronic hemodialysis patients: a national study. Am J Kidney Dis.

[15] Ketteler M, Westenfeld R, Schlieper G (2008;). Role of fetuin-A in calcification inhibition. Circulation.

[16] Guerin AP, London GM, Marchais SJ, Metivier F (2000;). Arterial stiffening and vascular calcifications in end-stage renal disease. Nephrol Dial Transplant.

[17] Aaltonen L, Koivuviita N, Seppänen M, Kröger H, Tong X, Löyttyniemi E (2022;). Association between bone mineral metabolism and vascular calcification in end-stage renal disease. BMC Nephrol.

[18] Asim M, Alkadi M, Hamad A, Othman M, Abuhelaiqa E, Fituri O (2020). Restructuring nephrology services to combat COVID-19 pandemic: report from a Middle Eastern country. World J Nephrol.

[19] Hamad AI, Asim M, Othman MA, Abuhelaiqa EA, Shurrab A, Elmadhoun IT (2022). National response to the COVID-19 Omicron variant crisis in the ambulatory hemodialysis service in the State of Qatar. Qatar Med J.

[20] Hamad A, Mefleh Al Halabi A, Ghazouani H, Habas EM, Mohamed Borham A, Mohamed Ismail S (2023). Time-series forecasting of hemodialysis population in the State of Qatar by 2030. Qatar Med J.

[21] Kristen L (2013;). Jablonski and Michel Chonchol. Vascular calcification in end-stage renal disease. Hemodial Int.

[22] Kidney Disease: Improving Global Outcomes (KDIGO) CKD-MBD Update Work Group (2017;). KDIGO 2017 Clinical Practice Guideline Update for the Diagnosis, Evaluation, Prevention, and Treatment of Chronic Kidney Disease-Mineral and Bone Disorder (CKD-MBD). Kidney Int Suppl (2011).

[23] London GM, Guerin AP, Marchais SJ, Métivier F, Pannier B, Adda H (2003;). Calcification in end-stage renal disease: impact on all-cause and cardiovascular mortality. Nephrol Dial Transplant.

[24] Toussaint ND, Pedagogos E, Lau KK, Heinze S, Becker GJ, Beavis J (2011;). Lateral lumbar X-ray assessment of abdominal aortic calcification in Australian haemodialysis patients. Nephrology.

[25] Watanabe S, Fujii H, Kono K, Watanabe K, Goto S, Nishi S (2020). Influence of oxidative stress on vascular calcification in the setting of coexisting chronic kidney disease and diabetes mellitus. Sci Rep.

[26] Chertow GM, Raggi P, Chasan-Taber S, Bommer J, Holzer H, Burke SK (2004;). Determinants of progressive vascular calcification in hemodialysis patients. Nephrol Dial Transplant.

[27] Phan O, Joki N (2022;). Vascular Calcification in Diabetic Kidney Disease. Kidney Dial.

[28] Ishimura E, Okuno S, Kitatani K, Kim M, Shoji T, t al (2002;). Different risk factors for peripheral vascular calcification between diabetic and non-diabetic haemodialysis patients – importance of glycaemic control. Diabetologia.

[29] Kimura K, Saika Y, Otani H, Fujii R, Mune M, Yukawa S (1999;). Factors associated with calcification of the abdominal aorta in hemodialysis patients. Kidney Int Suppl.

[30] Wang AY, Wang M, Woo J, Lam CW, Li PK, Lui SF (2003;). Cardiac valve calcification as an important predictor for allcause mortality and cardiovascular mortality in long-term peritoneal dialysis patients: a prospective study. J Am Soc Nephrol.

[31] Kauppila Ll, Polak JF, Cupples LA (1997;). New indices to classify location, severity and progression of calcific lesion in the abdominal aorta 25-years follow-up study. Atherosclerosis.

[32] Adragao T, Pires A, Lucas C, Birne R, Magalhaes L, Gonçalves M (2004;). A simple vascular calcification score predicts cardiovascular risk in haemodialysis patients. Nephrol Dial Transplant.

[33] García-Canton C, Bosch E, Ramírez A, Gonzalez Y, Auyanet I, Guerra R (2011;). Vascular calcification and 25-hydroxyvitamin D levels in non-dialysis patients with chronic kidney disease stages 4 and 5. Nephrol Dial Transplant.

[34] Górriz JL, Molina P, Cerverón MJ, Vila R, Bover J, Nieto J (2015;). Vascular calcifica¬tion in patients with nondialysis CKD over 3 years. Clin J Am Soc Nephrol.

[35] Honkanen E, Kauppila L, Wikstrom B, Rensma P, Krzesinski J, Aasarod K (2008;). Abdominal aortic calcification in dialysis patients: results of the CORD study. Nephrol Dial Transplant.

[36] Jean G, Bresson E, Terrat J, Vanel T, Hurot J, Lorriaux C (2009;). Peripheral vascular calcification in long-haemodialysis patients: associated factors and survival consequences. Nephrol Dial Transplant.

[37] SciELO Argentina (2017;). Medicina (B. Aires) vol.77 no.3 Ciudad Autónoma de Buenos Aires jun.

[38] London GM, Guerin AP, Verbeke FH, Pannier B, Boutouyrie P, Marchais SJ (2007;). Metivier F: Mineral metabolism and arterial functions in end-stage renal disease: Potential role of 25-hydroxyvitamin D deficiency. J Am Soc Nephrol.

